# CX3CL1 promotes M1 macrophage polarization and osteoclast differentiation through NF-κB signaling pathway in ankylosing spondylitis in vitro

**DOI:** 10.1186/s12967-023-04449-0

**Published:** 2023-08-25

**Authors:** Xinzhe Feng, Shanbang Zhu, Junjie Qiao, Zhou Ji, Bole Zhou, Weidong Xu

**Affiliations:** 1 Department of Joint Bone Disease Surgery, Changhai Hospital, Naval Medical University, 168 Changhai Road, Shanghai, 200433 China; 2 Department of Orthopaedics, Affiliated Jinling Hospital, Medical School of Nanjing University, No 305 Zhongshandonglu Road, Nanjing, 210002 China

**Keywords:** Ankylosing spondylitis, CX3CL1, Intestinal inflammation, Macrophages

## Abstract

**Background:**

Ankylosing spondylitis (AS) is an autoimmune disease with a genetic correlation and is characterized by inflammation in the axial skeleton and sacroiliac joints. Many AS patients also have inflammatory bowel diseases (IBD), but the underlying causes of intestinal inflammation and osteoporosis in AS are not well understood. CX3CL1, a protein involved in inflammation, has been found to be up-regulated in AS patients and AS-model mice.

**Methods:**

The authors investigated the effects of CX3CL1 on AS by studying its impact on macrophage polarization, inflammation factors, and osteoclast differentiation. Furthermore, the effects of inhibiting the NF-κB pathway and blocking CX3CL1 were assessed using BAY-117082 and anti-CX3CL1 mAb, respectively. AS model mice were used to evaluate the effects of anti-CX3CL1 mAb on limb thickness, spine rupture, and intestinal tissue damage.

**Results:**

The authors found that CX3CL1 increased the expression of M1-type macrophage markers and inflammation factors, and promoted osteoclast differentiation. This effect was mediated through the NF-κB signaling pathway. Inhibition of the NF-κB pathway prevented M1-type macrophage polarization, reduced inflammation levels, and inhibited osteoclast differentiation. Injection of anti-CX3CL1 mAb alleviated limb thickness, spine rupture, and intestinal tissue damage in AS model mice by inhibiting M1-type macrophage polarization and reducing intestinal tissue inflammation.

**Conclusions:**

The study demonstrated that up-regulated CX3CL1 promotes M1-type macrophage polarization and osteoclast differentiation through the NF-κB signaling pathway. Inhibition of this pathway and blocking CX3CL1 can alleviate inflammation and bone destruction in AS. These findings contribute to a better understanding of the pathogenesis of AS and provide a basis for clinical diagnosis and treatment.

**Supplementary Information:**

The online version contains supplementary material available at 10.1186/s12967-023-04449-0.

## Introduction

Ankylosing Spondylitis (AS) is an autoimmune disease, with a global incidence rate of 0.1–1.4% [1]. The etiology of AS is not fully understood, but most patients are influenced by genetic factors [2]. Early diagnosis and timely treatment are crucial in the treatment of AS. Traditional drug therapy is currently one of the main treatment methods for AS, improving the patient’s condition by suppressing immune responses and reducing inflammation symptoms [3, 4]. However, these drugs can only alleviate the inflammatory response and related symptoms, and cannot completely reverse the ossification process caused by the AS [5]. It is urgent to better understand the pathophysiological mechanisms of AS and find potential targets for the treatment of AS.

The gut microenvironment has become one of the hotspots in the research of autoimmune diseases. With the deepening of research on gut microbiology, gut microbiota, gut immune cells, and gut mucosal barrier, researchers have gradually realized that the gut microenvironment may regulate the occurrence and progression of autoimmune diseases [6, 7]. Studies have shown that inflammatory bowel disease (IBD) patients have imbalanced gut microbiota, abnormally activated gut immune cells, and damaged gut mucosal barrier, leading to intestinal inflammation [8, 9]. In addition, the abnormal activation of gut immune cells and imbalanced gut microbiota resulted in the progression of rheumatoid arthritis (RA) [10, 11]. However, we still unknow the role of imbalanced gut microbiota and abnormal gut immune cells in the occurrence and development of AS.

CX3CL1, encoded by *cx3cl1* gene, is a cytokine of a trend factor family to play a biological effect by combining with CX3CR1, a seven-transmembrane G protein-coupled receptor that is mainly expressed in immune cells, neurons, and the cardiovascular system [12–14]. In addition to its chemotactic function, CX3CL1 also has an adhesion molecule function, which promotes interactions between immune cells and neurons [15, 16]. Some studies have shown that CX3CL1 can promote immune cell infiltration and activation, thereby inhibiting tumorigenesis and regulating inflammatory response [17, 18]. CX3CR1 inhibitors have been shown to enhance the efficacy of immunotherapy in cancer treatment [19]. High expression of CX3CL1 may lead to excessive activation of immune cells and tissue damage in IBD and RA [20]. Macrophage-related chemokines, including CX3CL1 are closely associated with active inflammation of the sacroiliac joint in AS [21]. However, the role of aberrant expression of CX3CL1 in the pathogenesis of AS remains unclear.

In this study, through peripheral blood transcriptome sequencing, we founded the expression of CX3CL1 was significantly increased in the peripheral blood of AS patients with gut inflammation versus to healthy controls. Inhibiting CX3CL1 can prevent M1 macrophage polarization, reduce inflammation, and inhibit osteoclast differentiation by NF-κB signaling pathway in vivo and in vitro, thereby alleviating the progression of the disease. Our results are expected to provide new insights into the pathophysiological mechanisms of AS, providing a new theoretical basis for the early prevention and treatment of AS.

## Materials and methods

### Cell culture

THP-1 cells were purchased from Shanghai Meixuan Biotechnology (China). The cells were revived and cultured in RPMI-1640 medium containing 10% inactivated FBS (37 ℃, 5% CO_2_). The THP-1 cells were centrifuged and resuspended, and 2 × 10^5^ cells were seeded into each well of a 6-well plate.

### Sample collection

From February 2020 to April 2021, venous blood was collected from three healthy controls and three AS patients with concurrent IBD in the rheumatology and immunology department of Changhai Hospital, Naval Medical University. PBMCs were isolated from the blood samples for RNA sequencing (RNA-seq). All AS patients met the revised New York AS diagnostic criteria from 1984. Participants with other immune diseases, pregnant women, and those unable to provide blood samples were excluded from the study. All AS patients were HLA-B27 positive and did not have any other autoimmune diseases. In addition, mucosal tissue and peripheral blood were collected from 20 AS patients with concurrent IBD and from 10 healthy volunteers in the gastroenterology department. The collected tissue samples were stored in a − 80 ℃ freezer. All participants signed a written informed consent form and were approved by the ethics committee of Changhai Hospital, Naval Medical University.

### Enzyme-linked immunosorbent assay (ELISA)

Peripheral blood samples were collected and centrifuged at 1500 rpm for 15 min at 4 ℃. The supernatant was then transferred to an EP tube and left at room temperature for 1 h. The sample was then centrifuged at 3000 rpm for 10 min at 4 ℃. Samples were conducted according to the protocol of manufacturers (CST, USA). The standard curves were generated using the recombinant standards. The levels of cytokines were quantified using the serial dilutions of recombinant falling within the linear range.

### TRAP staining

THP-1 cells were seeded into a 48-well plate (6 × 10^5^ cells). For the experimental group, the culture medium was supplemented with RANKL (50 μg/ml) and M-CSF (50 μg/ml), and CX3CL1 was added for the CX3CL1 group. The control group was cultured in normal medium, with medium changes every 3 days. The THP-1 cells were stained on day 4, 7, and 10. Then follow the instructions on the TRAP assay kit (Sigma, Germany).

### Western blot

The cells were collected and lysed to extract total protein. The protein concentration was determined using a BCA protein quantification kit (Beyotime, China). Twenty micrograms of denatured protein were subjected to 10% SDS-PAGE and transferred to an NC membrane. The membrane was blocked with 5% skim milk for 1 h and then incubated with primary antibodies, including iNOS (ab178945, Abcam, USA), ACP5 (EPR21791, Abcam, USA), p-P65 (ab264271, Abcam, USA), p-IKKα/β (ab194528, Abcam, USA), p-IkBα/β (ab133462, Abcam, USA) and GAPDH (ab8245, Abcam, USA), at 4 ℃ overnight. After washing with 1 × TBST, the membrane was incubated with secondary antibodies at room temperature for 2 h. The membrane was washed again with 1 × TBST, and then visualized and exposed.

### Real-time quantitative PCR (RT-qPCR)

Total RNA was extracted from the cells using an RNA extraction kit (Promega, USA). Reverse transcription was performed using 30 ng of total RNA and 1 μmol/l circular primers. The reaction conditions were 85 ℃ for 5 min and 4 ℃ for 5 min. Real-time PCR was performed using the MX3000p real-time PCR detection system (Stratagene, USA) and the standard SYBR Green Assay protocol. Each 25 μl PCR reaction contained 2 μl of reverse transcription product, 1 × PCR Master Mix (Takara, Japan), 1.5 μmol/l forward primer, and 0.7 μmol/l reverse primer. The reaction was incubated at 95 ℃ for 10 s and then cycled 40 times at 95 ℃ for 5 s and 60℃ for 30 s. The relative expression of each gene was calculated using the 2^−ΔΔCt^ method and normalized to GAPDH. The primer sequences used for qPCR amplification were as follows Table 1.

**Table 1:** Target sequence of gene in RT-qPCR

Gene	Forward (5′ → 3′)	Reverse (5′ → 3′)
CX3CL1	ACCACGGTGTGACGAAATG	TGTTGATAGTGGATGAGCAAAGC
IL-17	TCCCACGAAATCCAGGATGC	GGATGTTCAGGTTGACCATCAC
TNF-α	GAGGCCAAGCCCTGGTATG	CGGGCCGATTGATCTCAGC
ACP-5	GGGAGATCTGTGAGCCAGTG	GGGAGCGGTCAGAGAATACG
iNOS	TCCAAGGTATCCTGGAGCGA	CAGGGACGGGAACTCCTCTA
Arg-1	ACTTAAAGAACAAGAGTGTGATGTG	GTCCACGTCTCTCAAGCCAA
GAPDH	AATGGGCAGCCGTTAGGAAA	GCGCCCAATACGACCAAATC

### Immunofluorescence

The formalin-fixed, paraffin-embedded intestinal pathological tissues were fixed with 4% paraformaldehyde (Beyotime, China) for 15 min. After being washed with phosphate buffered saline (PBS, Solarbio, China), the sections were subjected to 15 min treatment with Triton X-100 (Solarbio, China) and 1 h blocking using 5% goat serum (Absin, China). Then, the sections were incubated with primary antibodies against (CX3CL1, 1:200 and iNOS, 1:200) for 24 h. The next day, the sections were cultured with secondary antibodies Alexa Fluor 488-Labeled Goat anti-Mouse IgG (1:1000, Beyotime, China) and Alexa Fluor 488-Labeled Goat anti-Rabbit IgG (1:1000, Beyotime, China), followed by DAPI staining (Beyotime, China). The results were observed under a confocal microscope.

### Hematoxylin–eosin (HE) staining

After fixing the pathological tissue sections with 4% paraformaldehyde for 48 h, washing them with distilled water, dehydrating them until they were clear, sectioning them at a thickness of 4 μm, dewaxing them, and then rewatering them before staining them with HE. The sections are viewed under a microscope and the relevant data are photographed or recorded.

### Immunohistochemistry (IHC)

Lumbar sacral joint and ileum tissue slices were placed on a hot plate and baked at 65 ℃ for 1 h, followed by dewaxing and hydration according to the above procedure. IL-23 (ab45420, Abcam, USA) or anti-TNF-a (ab183218, Abcam, USA) antibody was added to the tissue with a pipette and incubated overnight at 4 ℃. After washing with PBS solution, the secondary antibody was added to the tissue with a pipette and incubated at room temperature for 1 h. DAB chromogenic solution was added to the tissue with a pipette. The chromogenic reaction was observed under a microscope.

### RNA library preparation and sequencing

RNA libraries were constructed using the TruSeq RNA Sample Prep Kit v2 (Illumina, Inc., San Diego, CA, USA). mRNA was purified using oligo(dT) magnetic beads and fragmented at 95 ℃ for 8 min using the fragment buffer in the TruSeq RNA Sample Prep Kit v2 (Illumina, Inc., San Diego, CA, USA). The first cDNA strand was synthesized using random oligonucleotides and SuperScript III reverse transcriptase (Invitrogen; Thermo Fisher Scientific Inc., Waltham, MA, USA) under the following conditions: 65 ℃ for 5 min, 4 ℃ for 2 min, 42 ℃ for 1 h, and 70 ℃ for 10 min. The second cDNA strand was synthesized by adding buffer, dNTP, RNase H (Invitrogen; Thermo Fisher Scientific Inc.), and DNA polymerase I (Invitrogen; Thermo Fisher Scientific Inc.) under the following conditions: 16 ℃ for 2.5 h and 70 ℃ for 10 min. The sequencing adapters were repaired and ligated using the NEB Next End Repair Module (New England Biolabs, Ipswich, MA, USA) after purification with the QIAQuick PCR Purification Kit (Qiagen, Inc., Valencia, CA, USA).

### Identification of differentially expressed genes (DEGs)

The quality of the sequencing data was evaluated using FastQC software (version 0.11.4). Cutadapt software (version 1.9.1) was used to remove low-quality reads, including sequences with a quality score < 20 and sequences with an N base rate > 10% of the original reads. TopHat 2.1.1 was used to align the cleaned reads to the human genome (GRCh38.p7 assembly). Cuffquant version 2.2.1 was used to obtain the counts and fragments per kilobase of transcript per million fragments mapped (FPKM) for each transcript mapped to each gene. DEGs were obtained using the DESeq2 package in R (version 3.6.3). The screening criteria for DEGs were |log2FC|≥ 1 and *p* < 0.05.

### Functional enrichment analysis

Gene Ontology (GO) enrichment analysis and Kyoto Encyclopedia of Genes and Genomes (KEGG) pathway enrichment analysis were performed using the ClusterProfiler package in R software. GO terms and KEGG pathways with p < 0.05 were considered significantly enriched. GO enrichment analysis divided into three parts: biological process (BP), cellular component (CC), and molecular function (MF).

### Immune cell infiltration analysis

Immune cell infiltration was analyzed using the "CIBERSORT" algorithm with the LM22 immune subtype. A gene-based deconvolution algorithm was used to quantify the relative scores of 22 human immune cell types using 547 marker genes.

### Construction of ankylosing spondylitis mouse model

The control group includes 10 BALB/c mice and the model group includes 20 SKG mice. The model group mice were injected with β-1,3-glucan solution (30 mg/kg, Sigma, USA) intraperitoneally once a week for 4 weeks. The control group mice were injected with an equal volume of saline. Then, the anti-CX3CL1 mAb group mice were injected with anti-CX3CL1 mAb (10 ng/ml) intravenously twice a week for 4 weeks. The morphology and color of all four paws of each mouse were observed daily, and the paw thickness of each mouse was measured twice a week and averaged to evaluate joint swelling.

### Micro-CT examination

The processed mouse spine was scanned using a Micro-CT machine (Bruker, Germany) for high-precision imaging. The scanning parameters were set to a source voltage of 60 kv, a source current of 166 μA, and a voxel resolution of 10 μm. After the scan was completed, we selected the distal end of the spine and the trabecular bone area of the backbone as the region of interest and performed 3D reconstruction. Using onboard software for analysis, we mainly analyzed parameters such as bone mineral density (BMD), trabecular number, trabecular thickness, trabecular separation, and trabecular pattern factor in the region of interest.

### Pathological scoring of the mouse spine intervertebral disc system

By calculating the average score of each individual subregion of the same and opposite sides of each animal's spine at each of the three spine levels, we obtained the total pathological score for each slice.

### Intestinal permeability

After 1 h of intragastric administration of DX-4000 FITC (600 mg/kg, Sigma, USA), peripheral blood was collected from the caudal vein in anesthetized mice. Add an equal volume of PBS solution to the separated serum sample, mix well, and then detect the fluorescence intensity of the sample using a spectrophotometer with an excitation wavelength of 485 nm and an emission wavelength of 53 nm. The corresponding concentration of DX-4000 FITC in the sample can be analyzed to reflect intestinal permeability in mice.

### Statistical analysis

The experimental data were statistically analyzed and graphed using R (version 3.6.3), GraphPad Prism 9.0, and SPSS 22.0 software. Values are presented as means ± standard error of mean (SEM), and t-test (comparison between two groups) or LSD analysis of variance was used when normal distribution and homogeneity of variance were satisfied (comparison between multiple groups). Dunnett's-T3 was used to compare uneven variance, *p* < 0.05 indicated statistical significance of the difference.

## Results

### DEGs and functional enrichment analysis

**Fig. 1:**
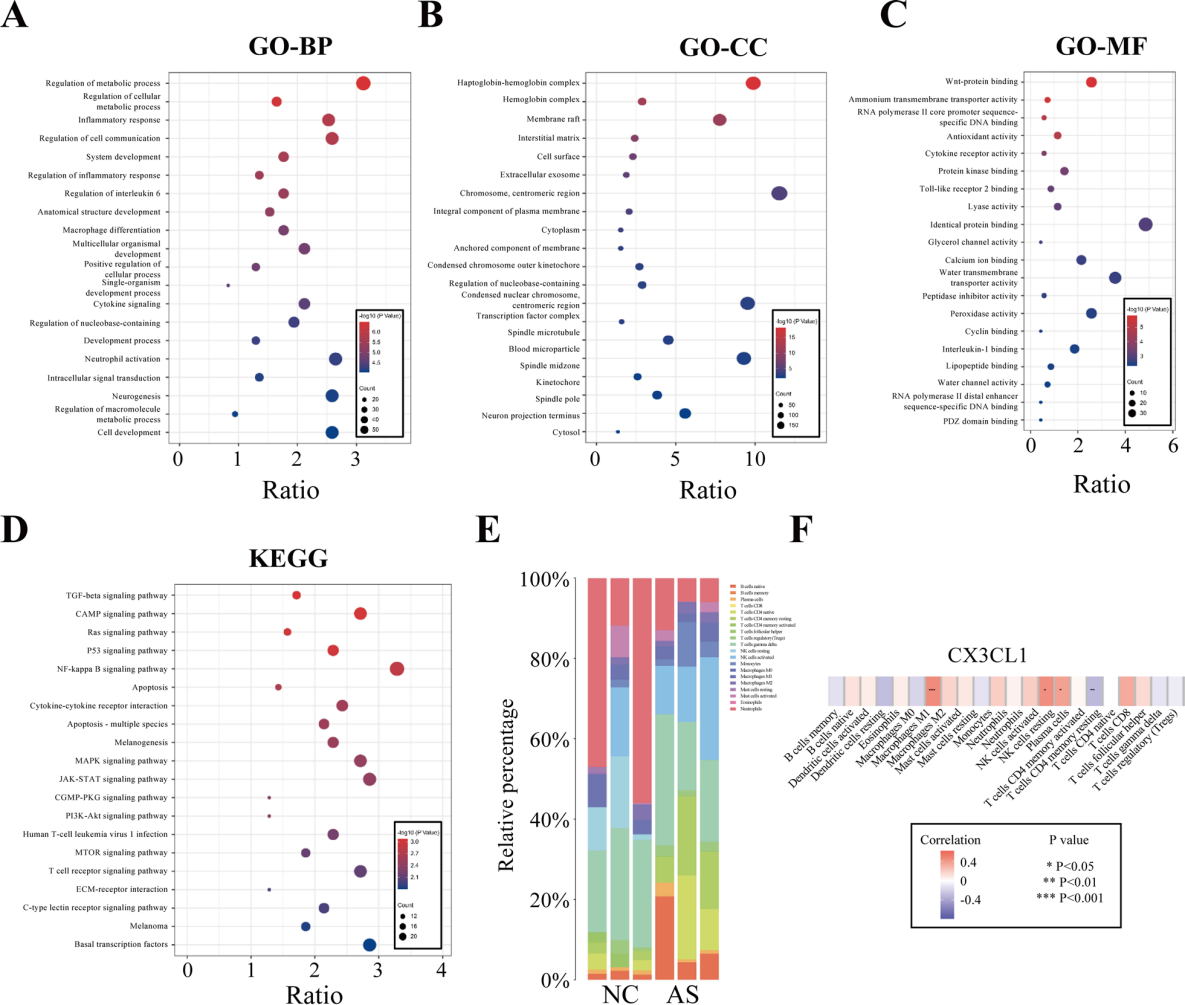
PBMCs expression profile analysis and functional enrichment analysis of DEGs. **A** biological processes in GO enrichment analysis; **B** cellular components; **C** molecular functions; **D** KEGG pathway enrichment analysis; **E** proportion of immune cells in each group in immune infiltration analysis; **F** correlation analysis between CX3CL1 and immune cells

Firstly, we apply the Deseq2 package in R software to analyze the DEG between the AS group and the NC group. The first 20 upregulated and downregulated genes with significant expression changes were preliminarily identified (Additional file 1: Figure S1 and Additional file 2: Table S1). Then, all 393 DEGs were performed GO and KEGG enrichment analysis with a threshold of p < 0.05 (Additional file 2: Tables S2 and S3). BP-related entries were mainly involved in inflammatory response, regulation of inflammatory response, regulation of interleukin-6, macrophage differentiation and cytokine signaling (Fig. 1A). CC-related entries mainly involved cytoplasm, cell surface, extracellular exosome and hemoglobin complex (Fig. 1B). MF-related entries mainly involved cytokine receptor activity, protein kinase binding, antioxidant activity and toll-like receptor 2 binding (Fig. 1C). The KEGG pathways showed that DEGs were mainly involved in Ras signaling pathway, NF-κB signaling pathway, cytokine-cytokine receptor interaction, p53 signaling pathway, and TGF-beta signaling pathway (Fig. 1D). We used the "cibersort" package to analyze the immune cell infiltration in AS and normal samples. Figure 1E showed the proportion of immune cell infiltration in each sample. In addition, CX3CL1 and M1-type macrophages showed the most significant positive correlation (Fig. 1F), indicating CX3CL1 may serve a key gene in the occurrence of AS.

### Expression of CX3CL1 is up-regulation in AS patients

**Fig. 2:**
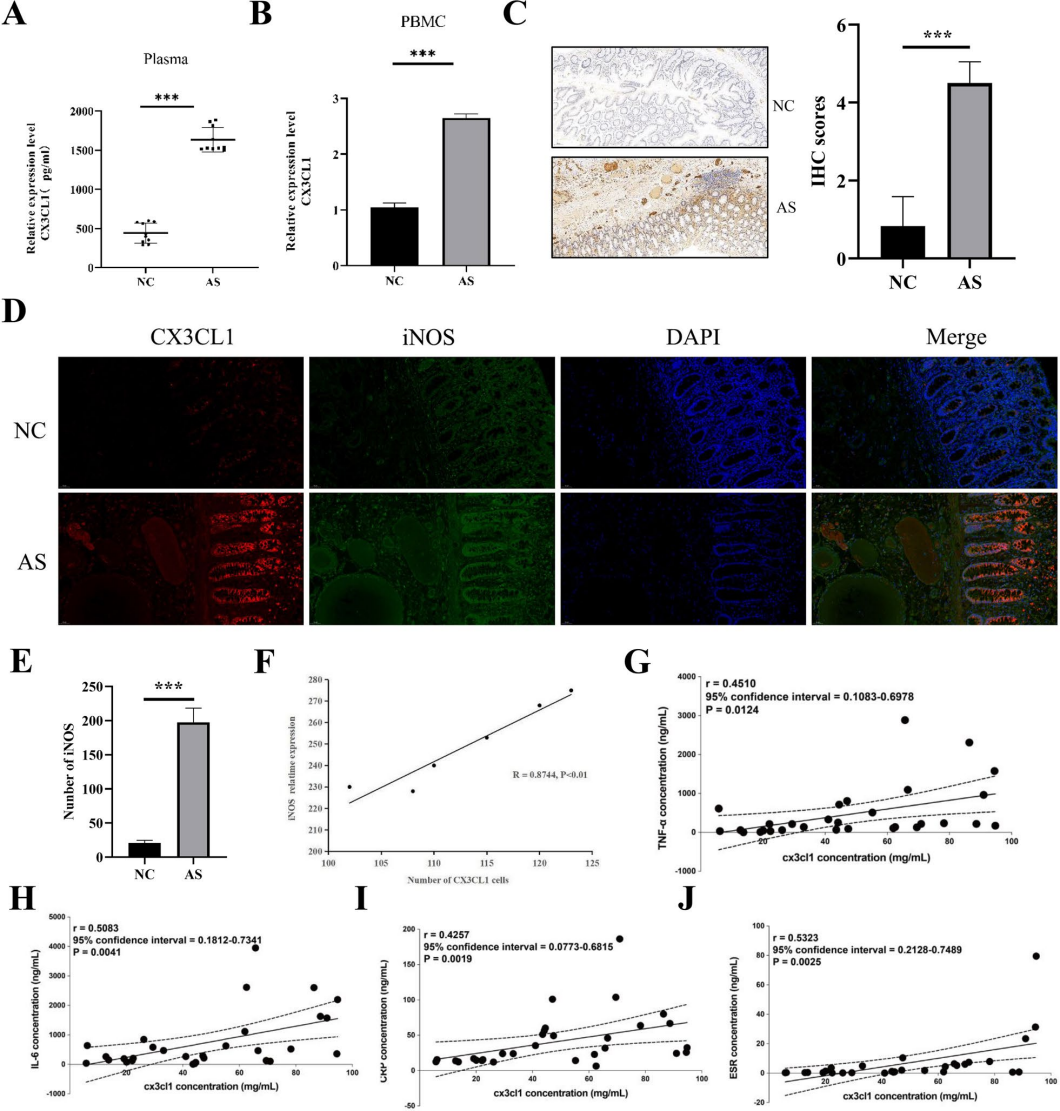
Expression analysis of CX3CL1 in AS patients. **A** expression levels of CX3CL1 in peripheral blood of AS and NC groups; **B** expression levels of CX3CL1 in PBMC of AS and NC groups; **C** expression levels of CX3CL1 in intestinal tissue of NC and AS groups; **D** co-localization of CX3CL1 and iNOS detected by immunofluorescence; **E** quantitative analysis of iNOS expression detected by immunofluorescence; **F** correlation analysis of CX3CL1 and iNOS; **G**–**J** correlation analysis of CX3CL1 expression in AS patients with TNF-α, IL-6, CRP, and ESR expression. n = 3, data are represented as mean ± SD, ^∗^*p* < 0.05 vs. Control, ^∗∗^*p* < 0.01 vs. Control, ^∗∗*^p < 0.001 vs. Control

Compared to the normal control blood samples, the expression of CX3CL1 were significantly increased in blood samples and peripheral blood mononuclear cells (PBMCs) from AS patients with IBD (Fig. 2A and B). Previous studies have reported that AS may disrupt the intestinal barrier and cause inflammation reactions such as IBD by regulating the composition of intestinal flora. Compared with the control group, the expression of CX3CL1 and the M1 macrophage marker (iNOS) were significantly increased in the intestinal tissue of AS patients with IBD (Fig. 2C–E). The expression level of CX3CL1 was positively correlated with iNOS in the intestinal tissue of AS patients (Fig. 2F). The expression of CX3CL1 in AS patients was also positively correlated with the expression of inflammatory factors (TNF-α, IL-6, CRP, ESR) (Fig. 2G–J).

### CX3CL1 promotes M1 macrophage polarization, cellular inflammation levels, and osteoclast differentiation

**Fig. 3:**
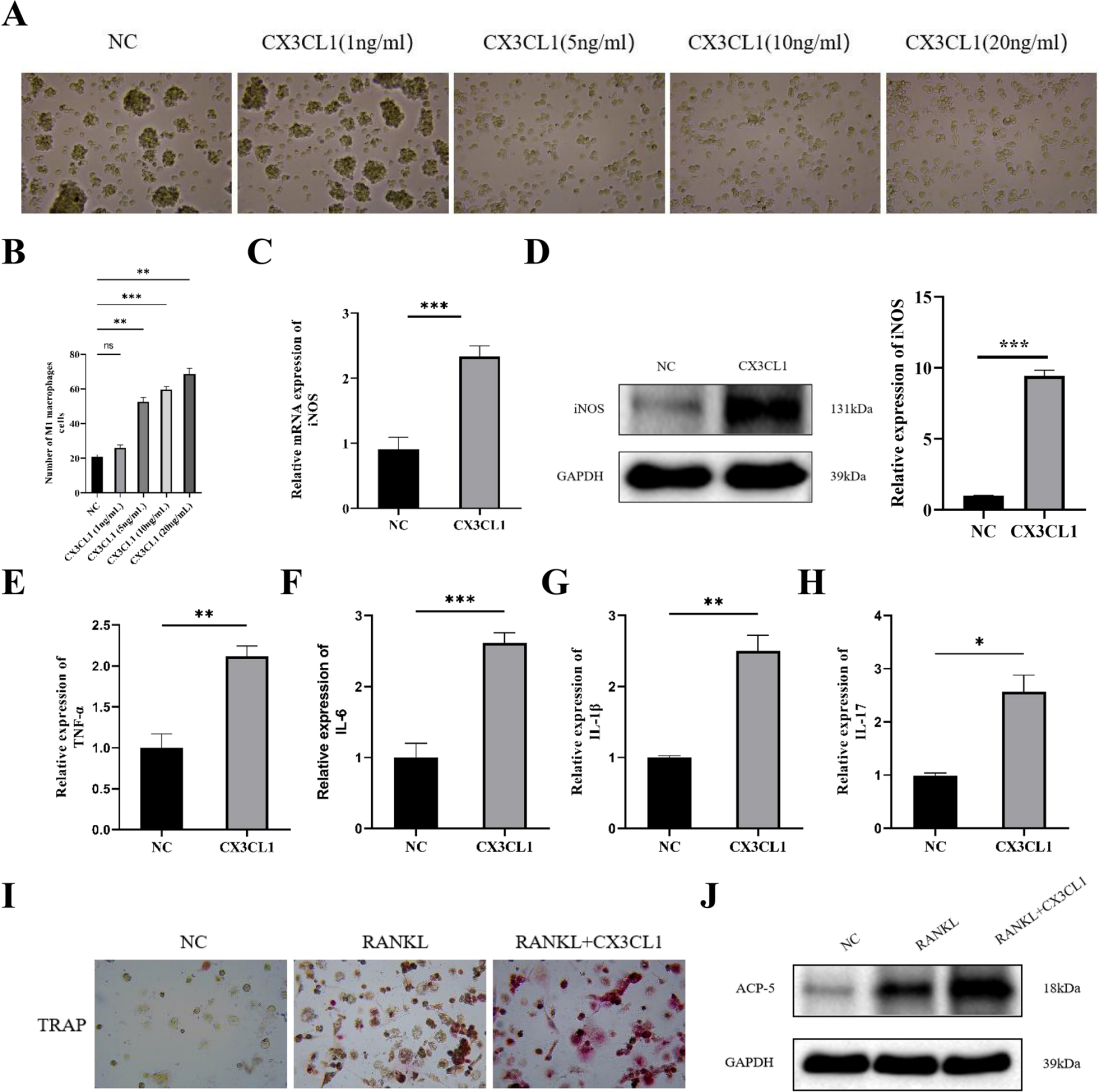
CX3CL1 promotes M1 macrophage polarization, cellular inflammation levels, and osteoclast differentiation. **A**, **B** determination of the optimal concentration of CX3CL1; **C** detection of iNOS expression by RT-qPCR; **D** expression of iNOS expression by Western blot. **E**–**H** ELISA detection of TNF-α, IL-6, IL-1β, and IL-17 expression in cell culture supernatant; **I** TRAP staining to investigate the effect of CX3CL1 on osteoclast differentiation; **J** detection of ACP-5 expression by Western blot. n = 3, data are represented as mean ± SD, ^∗^*p* < 0.05 vs. Control, ^∗∗^*p* < 0.01 vs. Control, ^∗∗*^p < 0.001 vs. Control

As a chemokine, CX3CL1 can promotes M1 macrophage polarization, and inhibition of CX3CL1 can cause macrophages to transition from M1 to M2 [22]. As shown in Fig. 3A and B, a moderate concentration of CX3CL1 (5 ng/ml), as the final concentration for further experiments, was the optimal method for inducing M1 macrophage polarization in THP-1 cell. RT-qPCR and Western blot results also showed that the expression of iNOS was significantly increased in the CX3CL1 group (Fig. 3C and D). Additionally, the expression of TNF-α, IL-6, IL-1β, and IL-17 were significantly increased in the CX3CL1 group from culture supernatants of THP-1 cell (Fig. 3E–H). Then, we explore whether adding CX3CL1 can promote osteoclast differentiation by TRAP staining, the results showed that CX3CL1 can promote osteoclast differentiation samliar as RANKL (Fig. 3I). The protein expression level of ACP-5 in the CX3CL1 + RANKL group was increased compared to the RANKL group (Fig. 3J).

### Differential expression analysis of CX3CL1 in M1 macrophages and osteoclasts

**Fig. 4:**
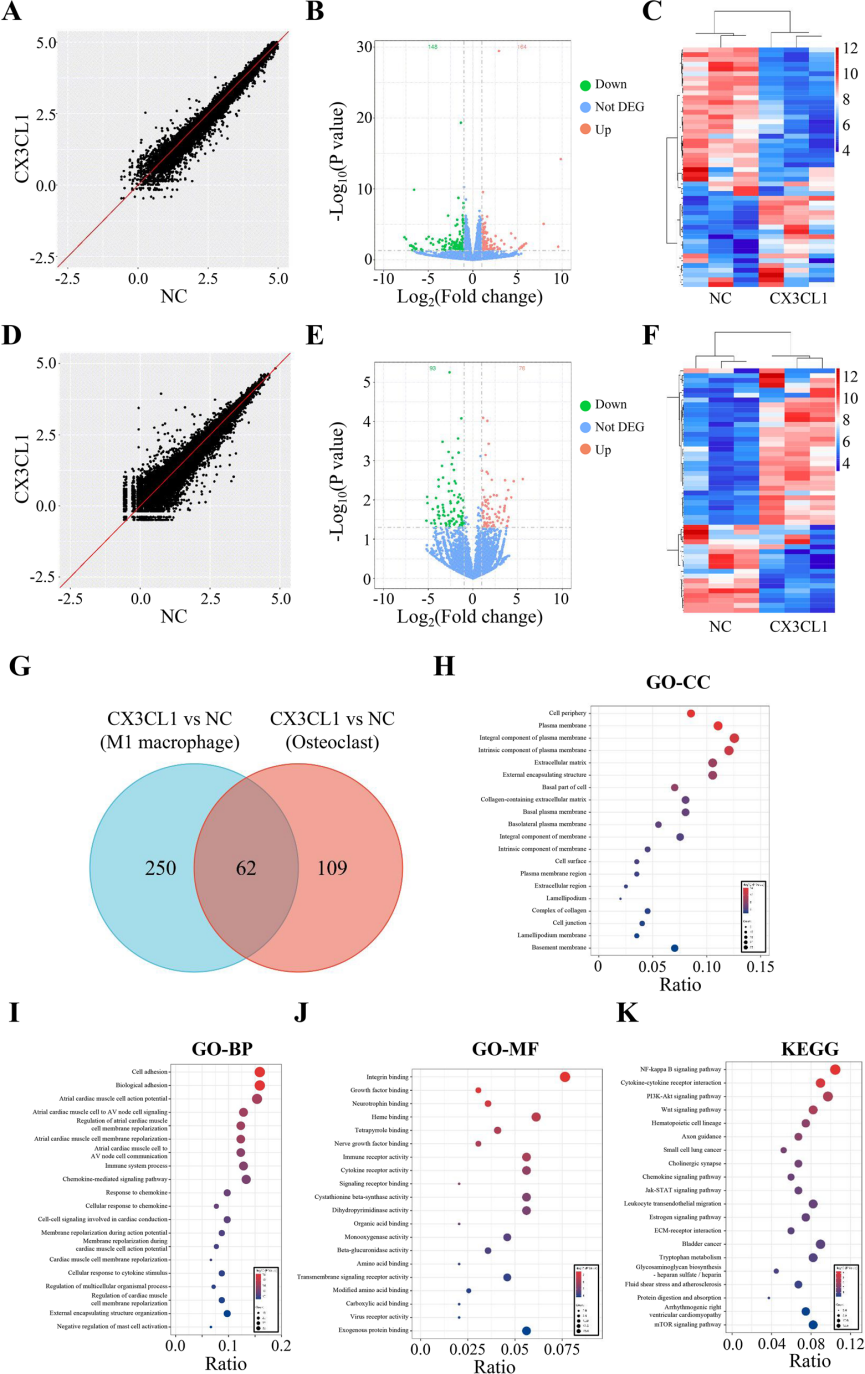
Differential expression analysis of CX3CL1 in M1 macrophages and osteoclasts. **A**–**C** Scatter plot (**A**), volcano plot (**B**), and cluster heatmap (**C**) of DEGs in CX3CL1 group and M1 macrophage group cells; **D**–**F** scatter plot (**D**), volcano plot (**E**), and cluster heatmap (**F**) of DEGs identified in CX3CL1 group and control group osteoblasts; **G** venn diagram of intersecting genes of DEGs between two groups; **H**–**J** GO enrichment analysis of intersecting genes in (**H**) cellular components, (**I**) biological processes, and (**J**) molecular functions; **K** KEGG pathway enrichment analysis of intersecting genes. n = 3, data are represented as mean ± SD, ^∗^*p* < 0.05 vs. Control, ^∗∗^*p* < 0.01 vs. Control, ^∗∗*^p < 0.001 vs. Control

Through RNA sequencing analysis, we screened 312 DEGs in the CX3CL1 group and M1 macrophage group (Additional file 2: Table S4, Fig. 4A–C), and 171 DEGs in the CX3CL1 group and osteoclast group (Additional file 2: Table S5, Fig. 4D–F). As shown in Fig. 4G, 62 intersection genes were identified, and top 10 upregulated and downregulated genes among the intersecting DEGs were shown in Additional file 2: Table S6. By GO and KEGG enrichment analysis, 186 GO terms and 47 KEGG pathways were explored with a threshold of p < 0.05. The results showed that intersecting DEGs are mainly involved in the NF-κB signaling pathway, Wnt signaling pathway, cytokine-cytokine receptor interaction, PI3K-Akt signaling pathway, and Jak-STAT signaling pathway (Fig. 4H–K, Additional file 2: Tables S7 and S8).

### CX3CL1 promotes M1 macrophage polarization, inflammation levels, and osteoclast differentiation through the NF-κB pathway

**Fig. 5:**
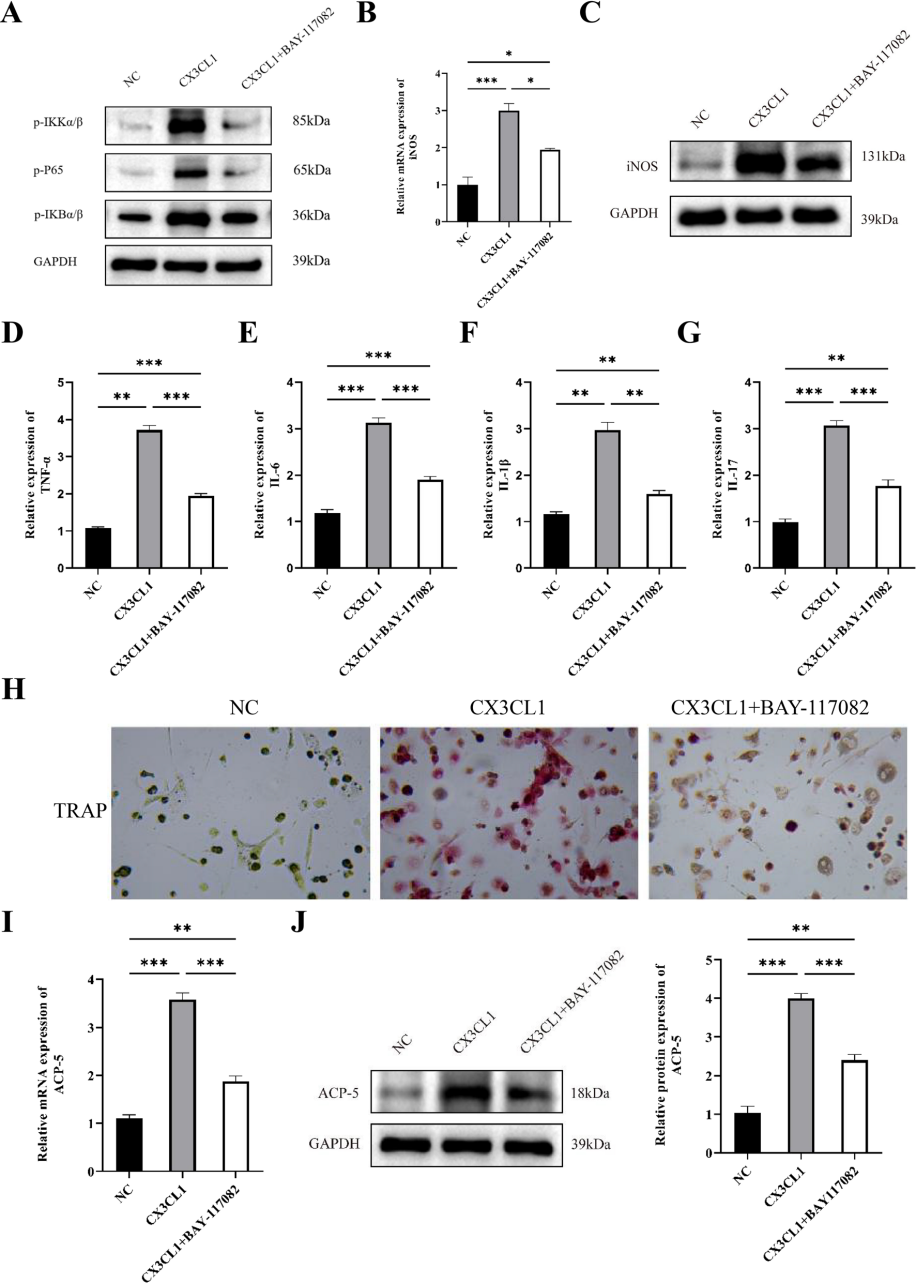
CX3CL1 promotes M1 macrophage polarization, inflammation levels, and osteoclast differentiation through the NF-κB pathway. **A** Western blot analysis of NF-κB signaling pathway-related protein expression; **B** RT-qPCR analysis of iNOS expression; **C** western blot analysis of iNOS expression; **D**–**G** ELISA analysis of TNF-α, IL-6, IL-1β, and IL-17 expression; **H** TRAP staining to detect the effect of NF-κB pathway inhibition on osteoclast differentiation; **I** RT-qPCR analysis of ACP-5 expression; **J** western blot analysis of ACP-5 expression. n = 3, data are represented as mean ± SD, ^∗^*p* < 0.05 vs. Control, ^∗∗^*p* < 0.01 vs. Control, ^∗∗*^p < 0.001 vs. Control

From the KEGG pathway enrichment analysis, the NF-κB signaling pathway is the most significant pathway. Therefore, we investigated the effect of CX3CL1 on the NF-κB signaling pathway by Western blot. The results showed that CX3CL1 significantly increased the expression levels of p-P65, p-IKKα/β, and p-IkBα/β in the cells (Fig. 5A). BAY-117082 is an NF-κB pathway inhibitor that can inhibit p65 nuclear translocation and DNA binding activity [23]. According to previous research [24], we treated cells with 10 µM BAY-117082 (Med Chem Express) at 37 ℃ for 1 h to inhibit NF-κB pathway. Compared with the CX3CL1 group, the level of iNOS was significantly decreased in the CX3CL1+ BAY-117082 group (Fig. 5B, C). ELISA results showed that the expression of TNF-α, IL-6, IL-1β, and IL-17 was significantly decreased in the CX3CL1 + BAY-117082 group (Fig. 5D–G), indicating CX3CL1 can promote inflammatory response by NF-κB pathway. TRAP staining and the expression of ACP-5 was significantly decreased in the CX3CL1+ BAY-117082 group versus to CX3CL1 group (Fig. 5H–J).

### Expression of CX3CL1 is upregulated in AS model mice

**Fig. 6:**
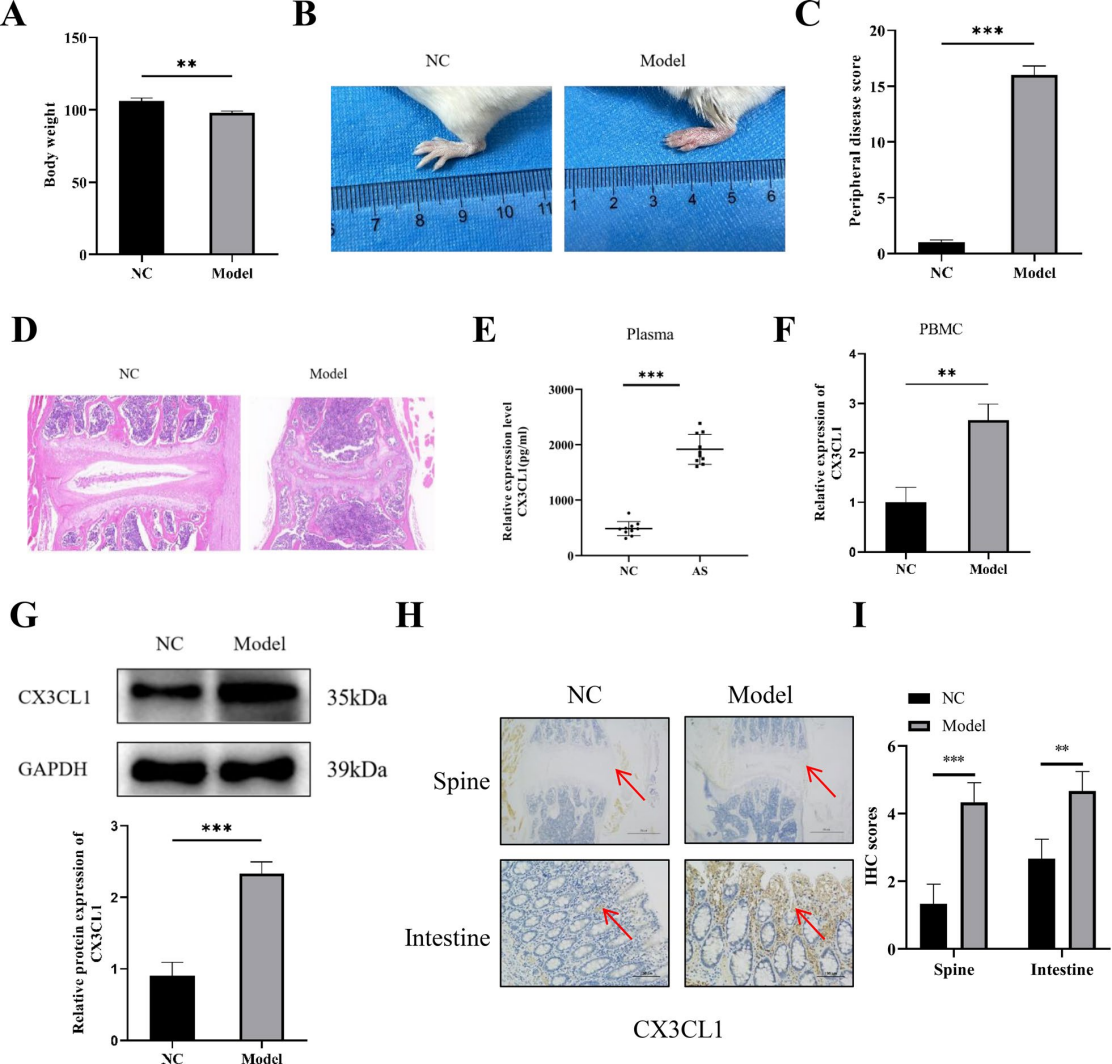
Expression of CX3CL1 in AS-model mice. **A** Comparison of body weight between AS mice and control mice; **B** comparison of limb thickness between AS mice and control mice; **C** peripheral joint scores of AS mice and control mice; **D** histological staining of spinal joint tissue in AS mice and control mice; **E** expression levels of CX3CL1 in peripheral blood of mice; **F** expression levels of CX3CL1 in PBMCs of mice; **G** western blot analysis of CX3CL1 expression in spinal joint tissue of mice; **H** IHC analysis of CX3CL1 expression in spine and intestinal tissue of mice; **I** quantitative analysis of IHC analysis of CX3CL1 expression in intestinal tissue of mice. n = 3, data are represented as mean ± SD, ^∗^*p* < 0.05 vs. Control, ^∗∗^*p* < 0.01 vs. Control, ^∗∗*^p < 0.001 vs. Control

During the injection of β-1,3-glucan, the model group of mice had poor mental state, restlessness, decreased food intake, increased water intake, dull and wet fur, and poor glossiness (Additional file 2: Table S9). Compared with the control group, mice in the model group lost weight and increased the thickness of limbs (Figs. 6A, B). By peripheral joint score of the mice, the average scores of the model group of mice (16 ± 0.8) were higher than the control group of mice (0.8 ± 0.3) (Fig. 6C). The HE results of spinal pathology sections showed that the model group of mice had a narrower intervertebral disc space, sparse vertebral endplates and cortical bone loss, collapsed intervertebral disc height, and vertebral osteophyte formation (Fig. 6D).

The expression of CX3CL1 in the peripheral blood and PBMC of the model group of mice were significantly up-regulated (Fig. 6E, F). In addition, we also detected the expression level of CX3CL1 in mouse spinal and intestinal tissues by Western blot and IHC. The results showed that the expression of CX3CL1 in the model group of mice was significantly increased in the spine and intestinal tissues (Fig. 6G–I). Taken together, increased CX3CL1 may promote the occurrence and progression of AS.

### Inhibiting CX3CL1 alleviates spinal damage, intestinal inflammation, and M1 macrophage polarization in AS model mice

**Fig. 7:**
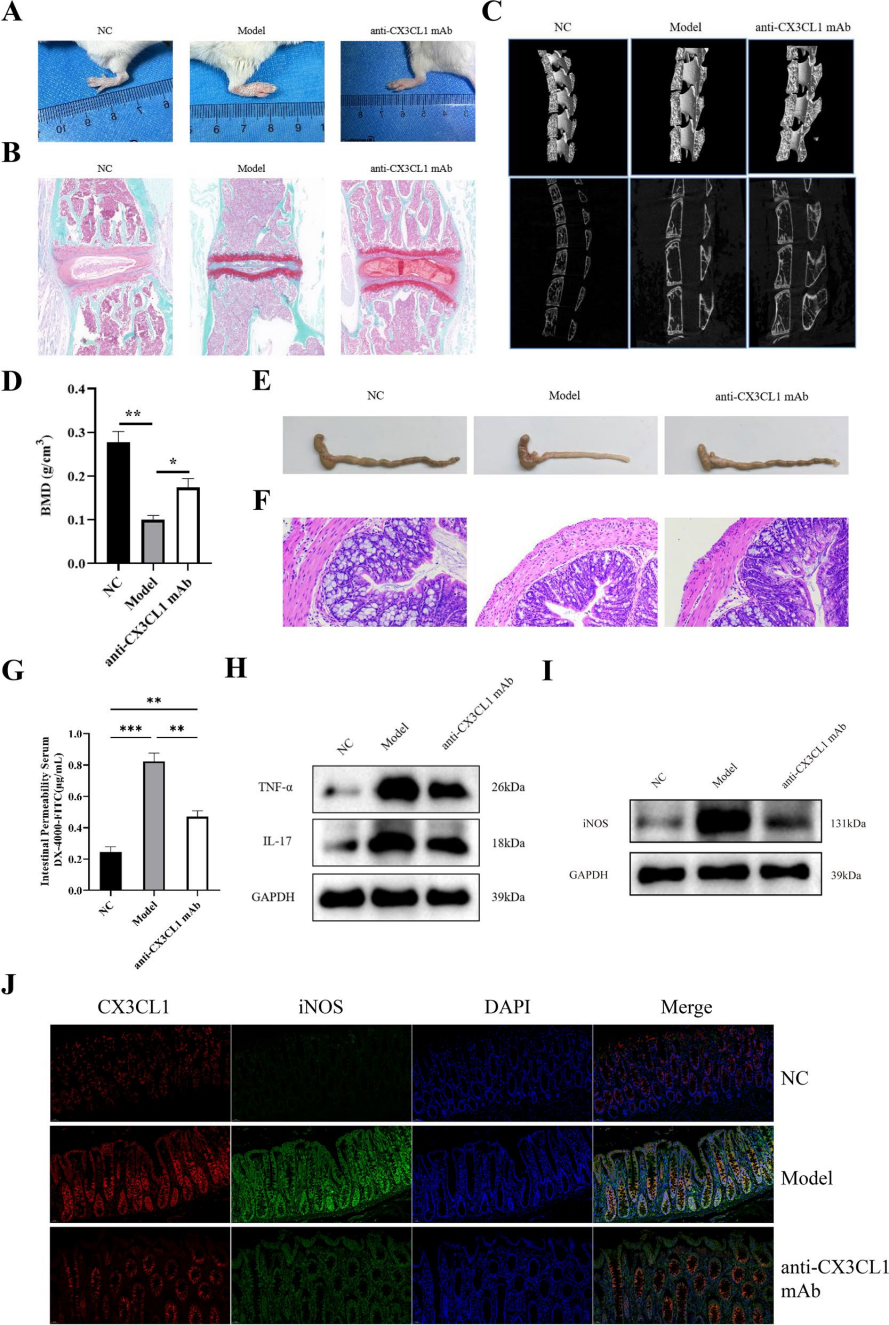
Inhibiting CX3CL1 alleviates spinal damage, intestinal inflammation, and M1 macrophage polarization in AS-model mice. **A** Thickness of limbs in mice; **B** safranin O-Fast Green staining of intervertebral discs in the spine; **C** MicroCT of the spine; **D** bone density values; **E** intestinal length; **F** HE staining of intestinal tissue; **G** intestinal permeability test; **H**, **I** western blot detection of TNF-α, IL-17, and iNOS expression in mouse intestines; **J** immunofluorescence detection of CX3CL1 and iNOS expression in mice intestines. n = 3, data are represented as mean ± SD, ^∗^*p* < 0.05 vs. Control, ^∗∗^*p* < 0.01 vs. Control, ^∗∗*^p < 0.001 vs. Control

To explore the possibility of treating AS by inhibiting CX3CL1, we randomly selected 10 AS model mice and intravenously injected anti-CX3CL1 mAb, which can inhibit CX3CL1 expression and reduce CX3CL1-mediated inflammatory responses [25]. The results showed that the anti-CX3CL1 mAb group of mice had a reduced paw thickness swelling and decreased intervertebral disc narrowing and damage (Fig. 7A, B). Whereafter, we observed the changes in mouse spinal imaging by MicroCT, the anti-CX3CL1 mAb group of mice had increased bone mass and bone formation, and the number, morphology distribution, and connection status of bone trabeculae had a certain degree of recovery (Fig. 7C). The anti-CX3CL1 mAb group of mice showed a significant increased BMD value, which is the gold standard for diagnosing osteoporosis (Fig. 7D). The length of intestine was increased (Fig. 7E) and the integrity of ileal tissue was restored (Fig. 7F) in the anti-CX3CL1 mAb group of mice. Additionally, the level of DX-4000-FITC in the serum of the anti-CX3CL1 mAb group of mice was significantly reduced, indicating that the intestinal permeability of mice had been restored (Fig. 7G). The expression of IL-17, TNF-α and iNOS was inhibited by injection of anti-CX3CL1 mAb group (Fig. 7H–J), indicating anti-CX3CL1 mAb can obviously alleviate inflammatory response of AS mice.

## Discussion

**Fig. 8:**
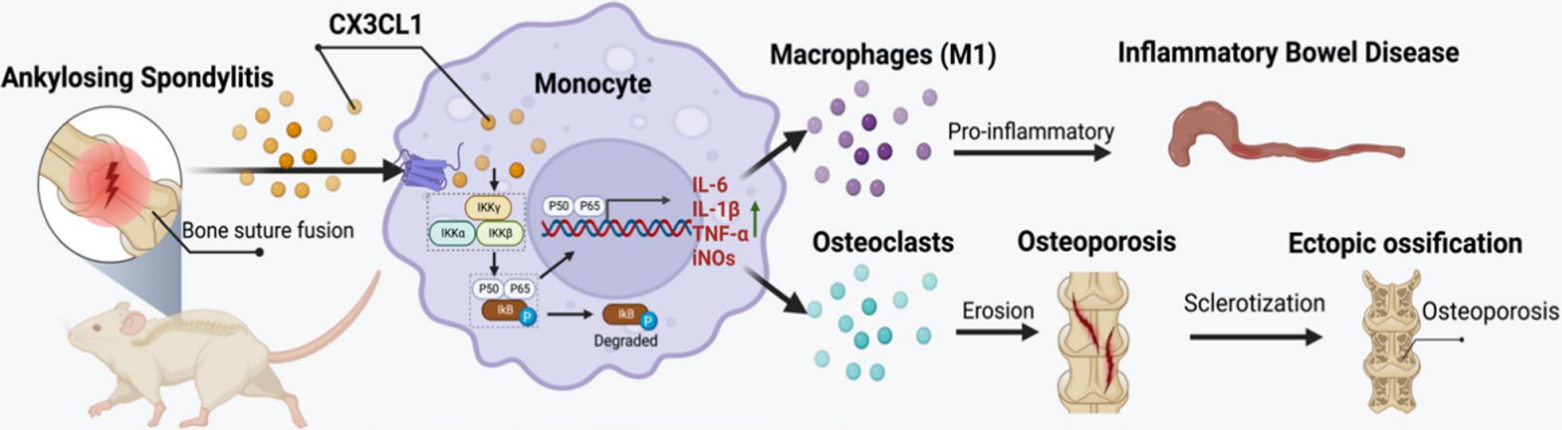
Molecular mechanism of CX3CL1 mediating AS intestinal inflammation and osteoporosis

As an autoimmune disease with significant genetic correlation, AS often presents with extra-articular manifestations such as uveitis, psoriasis, and IBD [1, 26, 27]. IBD is a chronic idiopathic disease characterized by intestinal mucosal lesions, which also regulated by the immune system [28, 29]. Previous studies have found that about 10% of AS patients will develop intestinal diseases with IBD features, and up to one-third of IBD patients may develop AS [30]. In addition, 40–60% of AS patients can observe subclinical intestinal inflammation under the naked eye and microscope. However, the potential pathogenesis of combined intestinal inflammation in AS patients have been unelucidated. This study has revealed a significant molecular mechanism in the investigation of the pathophysiology of AS in relation to intestinal inflammation and osteoporosis (Fig. 8).

In this study, we identified 393 DEGs (355 up-regulated genes and 38 down-regulated genes) in the peripheral blood of AS patients with IBD by RNA-seq. The GO and KEGG enrichment results showed that DEGs mainly participated in inflammation-related biological processes (inflammatory response, macrophage differentiation, cytokine signaling, NF-κB pathway and TGF-beta signaling pathway), which suggests that inflammation is an important factor in the pathogenesis of AS. CX3CL1 is the most significantly up-regulated gene in AS patients (log_2_ FC = 9.65). CX3CL1 can recruit macrophages to the peripheral nervous system, activate macrophages, and induce the release of various pro-inflammatory cytokines, exacerbating pathological pain [31]. A clinical study confirmed the existence of overlapping susceptibility loci between AS and IBD [32]. Consistent with RNA-seq results, the expression of CX3CL1 in the peripheral blood and intestinal mucosal of AS patients with IBD was significantly increased compared to the NC group by RT-qPCR and IHC. Furthermore, we found that CX3CL1 was positively correlated with M1 macrophage marker (iNOS) and inflammatory factors (TNF-α, IL-6, CRP, ESR) in AS patients. This proves that CX3CL1 may plays a pro-inflammatory role in the pathogenesis of AS by activating M1 macrophages.

THP-1 cells can simulate the role and response of macrophages in human pathophysiological processes such as immunity, inflammation and tumor [33]. Therefore, THP-1 cells were selected to simulate the process of macrophage polarization in vitro. CX3CL1 can increase the expression level of iNOS and inflammatory factors (TNF-α, IL-6, IL-1β and IL-17) in the THP-1 cell, which further indicated that CX3CL1 promote the inflammatory response by activating M1 macrophages in the pathogenesis of AS. Multiple studies have also shown that CX3CL1 is involved in the progression of osteoporosis, and the imbalance of bone remodeling caused by abnormal osteoclast differentiation is a key factor leading to osteoporosis [34]. RANKL is an important factor regulating osteoclast differentiation and ACP-5 is a recognized marker of osteoclasts [35–37]. In this study, we found that the effect of CX3CL1 was similar to the RANKL, promoting osteoclast differentiation and increasing the mRNA and protein expression levels of ACP-5 in THP-1 cells. These results suggest that CX3CL1 may be involved in the progression of osteoporosis by promoting osteoclast differentiation.

Then, we performed RNA-seq on CX3CL1 and control group M1 macrophages, as well as CX3CL1 and control group osteoclasts, in which 62 intersection genes were identified between the two groups. In order to explore the common molecular mechanisms that lead to M1 macrophage polarization and osteoclast differentiation, we performed KEGG pathway analysis, which showed that the NF-κB signaling pathway was the most significantly enriched pathway. NF-κB contains a transcription factor family, including RELA, RELB, NF-κB1, NF-κB2, and IκBα, which are closely related to the immune system [38]. Meanwhile, as one of the transcription factors of many pro-inflammatory cytokines, NF-κB is involved in the recognition of inflammation and the production of pro-inflammatory cytokines, and induces the release of IL-1β, TNF-α, IL-6 and other pro-inflammatory cytokines through pro-inflammatory cytokine receptors [39, 40]. By activating the NF-κB signaling pathway, M1 macrophage polarization and inflammatory reactions can be promoted [41]. Studies have shown that the expression of various inflammatory mediators and cytokines during M1 macrophage polarization is related to the activation and regulation of the NF-κB signaling pathway. The expression of inflammatory mediators such as IL-1β and TNF-α is regulated by the NF-κB signaling pathway, and the nuclear translocation of the p65/p50 complex increases the production of pro-inflammatory cytokines and promotes M1 macrophage polarization [42]. Besides, the NF-κB signaling pathway regulates osteoclast differentiation [43]. Under external environmental stimulation, the NF-κB signaling pathway is activated, inducing the expression of multiple osteoclast-specific genes in the nucleus, including MMP-9, TRAP, CTSK [44]. In addition, the NF-κB signaling pathway can also promote osteoclast differentiation by regulating the Wnt signaling pathway [45]. In this study, we chose BAY-117082 as an inhibitor of the NF-κB signaling pathway. Inhibiting the NF-κB signaling pathway reduced the effects of CX3CL1 on M1 macrophage polarization and osteoclast differentiation, suggesting that CX3CL1 exerts these biological effects through the NF-κB signaling pathway. Injection of anti-CX3CL1 mAb alleviated the increase in limb thickness, spine rupture and intestinal tissue damage in AS model mice. Inhibition of CX3CL1 can prevent the polarization of M1-type macrophages and reduce the level of intestinal tissue inflammation.

The relationship between CX3CL1 and the NF-κB pathway is crucial in immune responses and inflammatory reactions [46]. CX3CL1 is a chemokine that can regulate the migration and activation of immune cells. On the other hand, NF-κB is a transcription factor that can control the expression of many genes related to inflammatory responses. In certain disease states, such as inflammation, the activity of CX3CL1 and NF-κB may increase, leading to an excessive inflammatory response that can harm the body [47]. Therefore, theoretically, by inhibiting the CX3CL1 and NF-κB pathway, it may help alleviate the symptoms of these diseases. However, the CX3CL1 and NF-κB pathway also play a vital role in normal immune responses and inflammatory reactions. If these two pathways are completely inhibited, it may affect normal immune function, making the body more susceptible to infection. Therefore, the use of this strategy for treatment must be very cautious. In addition, the gut microbiota is involved in various physiological activities in the human body, and changes in the microbiota may affect the regulatory mechanism of CX3CL1 and also affect the host immune response, which is a direction worth exploring.

## Conclusion

This study found that CX3CL1 expression is significantly increased in AS, indicating that CX3CL1 plays an important role in the pathogenesis of AS. CX3CL1 may promote M1 macrophage polarization and osteoclast differentiation through the NF-κB signaling pathway. Inhibiting CX3CL1 can prevent M1 macrophage polarization, reduce inflammation, and inhibit osteoclast differentiation in vivo and in vitro, thereby alleviating the progression of the disease.

### Supplementary Information

**Additional file 1: Figure S1. A** Scatter plot of DEGs; **B** volcano plot of DEGs; **C** gene clustering heatmap.

**Additional file 2: Table S1.** Differential expression genes between AS group and NC group (upregulated/downregulated Top10 gene). **Table S2.** Significantly Enriched GO Entries (Top 20). **Table S3.** Significantly enriched KEGG pathway (Top20). **Table S4.** Differential expression genes of M1 type macrophages between the CX3CL1 group and the control group (upregulated/downregulated Top10 gene). **Table S5.** Differential expression genes between CX3CL1 group and control group osteoclast (up/down regulation of Top10 gene). **Table S6.** Intersection differentially expressed genes differentially expressed genes (upregulated/downregulated Top10 gene). **Table S7.** Significantly enriched GO pathway (Top30). **Table S8.** Significantly enriched KEGG pathway (Top20). **Table S9.** Changes in General Conditions of Mice (n=10).

## Data Availability

The datasets used and analyzed during the current study are available from the corresponding author on reasonable request.
