# Supplementary figures and images for: CX3CL1 promotes M1 macrophage polarization and osteoclast differentiation through NF-κB signaling pathway in ankylosing spondylitis in vitro
Source: J Transl Med. 2023 Aug 25;21:573. doi: 10.1186/s12967-023-04449-0 (PMC10463543; doi:10.1186/s12967-023-04449-0)

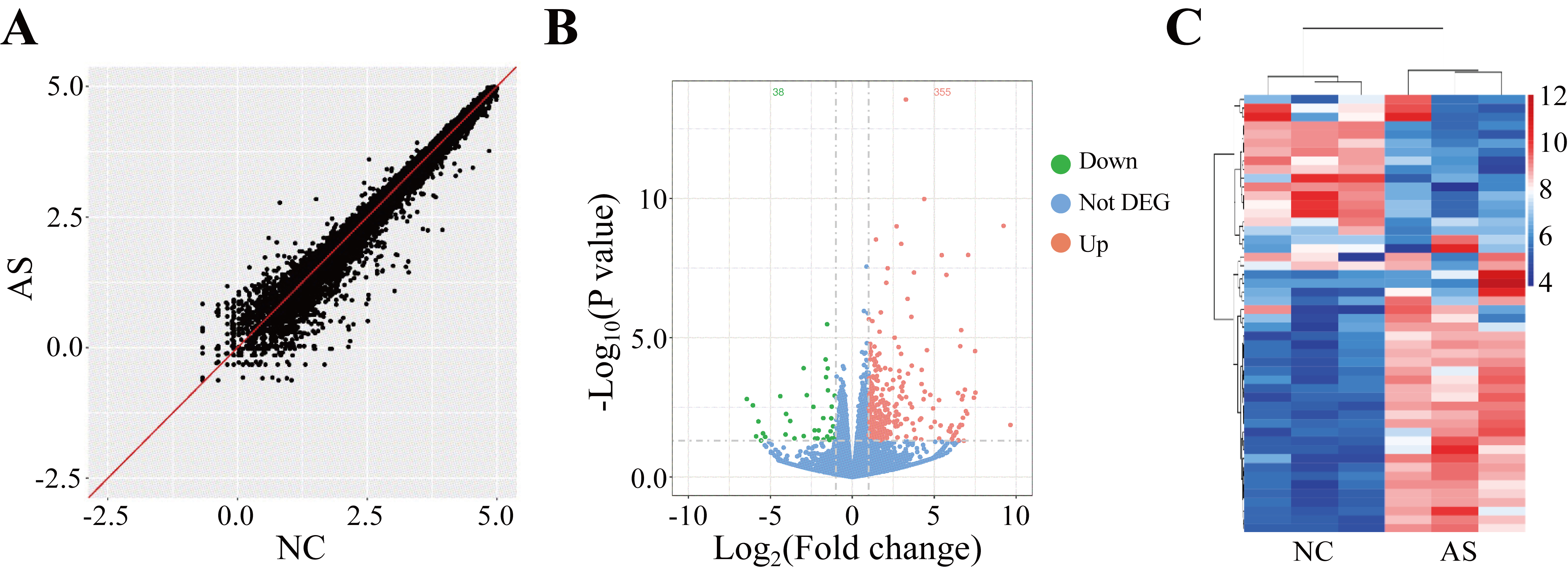

Supplement: Supplementary file 1 — Additional file 1: Figure S1. A Scatter plot of DEGs; B volcano plot of DEGs; C gene clustering heatmap. [file 12967_2023_4449_MOESM1_ESM.jpg]
