# Supplementary material for: CX3CL1 promotes M1 macrophage polarization and osteoclast differentiation through NF-κB signaling pathway in ankylosing spondylitis in vitro
Source: J Transl Med. 2023 Aug 25;21:573. doi: 10.1186/s12967-023-04449-0 (PMC10463543; doi:10.1186/s12967-023-04449-0)
Supplement: Supplementary file 2 — Additional file 2: Table S1. Differential expression genes between AS group and NC group (upregulated/downregulated Top10 gene). Table S2. Significantly Enriched GO Entries (Top 20). Table S3. Significantly enriched KEGG pathway (Top20). Table S4. Differential expression genes of M1 type macrophages between the CX3CL1 group and the control group (upregulated/downregulated Top10 gene). Table S5. Differential expression genes between CX3CL1 group and control group osteoclast (up/down regulation of Top10 gene). Table S6. Intersection differentially expressed genes differentially expressed genes (upregulated/downregulated Top10 gene). Table S7. Significantly enriched GO pathway (Top30). Table S8. Significantly enriched KEGG pathway (Top20). Table S9. Changes in General Conditions of Mice (n=10). [file 12967_2023_4449_MOESM2_ESM.docx]

**Table S1 Differential expression genes between AS group and NC group (upregulated/downregulated Top10 gene)**

| **Gene ID** | **Gene Symbol** | **log_2_FC** | **p value** | **Type** |
| --- | --- | --- | --- | --- |
| 6376 | CX3CL1 | 9.65 | 5.34E-06 | Up |
| 1050 | CEBPA | 8.23 | 2.02E-05 | Up |
| 339665 | SLC35E4 | 7.51 | 7.33E-04 | Up |
| 25803 | SPDEF | 7.49 | 9.65E-04 | Up |
| 3569 | IL6 | 7.42 | 1.23E-03 | Up |
| 3037 | HAS2 | 7.07 | 1.64E-03 | Up |
| 7069 | THRSP | 7.01 | 7.49E-03 | Up |
| 234564 | CES1F | 6.93 | 1.35E-02 | Up |
| 56729 | RETN | 6.87 | 1.36E-02 | Up |
| 4852 | NPY | 6.80 | 1.63E-02 | Up |
| 300551 | OLR1226 | -6.47 | 1.24E-04 | Down |
| 9889 | ZBED4 | -6.47 | 1.16E-03 | Down |
| 217698 | ACOT5 | -6.44 | 1.26E-03 | Down |
| 4151 | MB | -6.06 | 1.59E-03 | Down |
| 347741 | OTOP3 | -5.87 | 2.68E-03 | Down |
| 9248 | GPR50 | -5.73 | 3.03E-03 | Down |
| 502891 | VOM2R51 | -5.56 | 5.44E-03 | Down |
| 51305 | KCNK9 | -5.45 | 9.76E-03 | Down |
| 343413 | FCRL6 | -5.34 | 1.01E-02 | Down |
| 405353 | OLR1108 | -5.31 | 2.11E-02 | Down |

**Table S2 Significantly Enriched GO Entries (Top 20)**

| **GO Term** | **GO Category** | **p value** | **FDR** |
| --- | --- | --- | --- |
| hemoglobin complex | CC | 7.22E-19 | 1.03E-03 |
| membrane raft | CC | 1.10E-12 | 1.03E-03 |
| interstitial matrix | CC | 8.59E-12 | 1.03E-03 |
| cell surface | CC | 2.42E-09 | 3.89E-03 |
| extracellular exosome | CC | 1.83E-07 | 4.84E-03 |
| regulation of metabolic process | BP | 3.38E-07 | 1.21E-03 |
| regulation of cellular metabolic process | BP | 4.46E-07 | 1.21E-03 |
| inflammatory response | BP | 1.56E-06 | 2.11E-03 |
| regulation of cell communication | BP | 1.56E-06 | 2.11E-03 |
| Wnt-protein binding | MF | 1.65E-06 | 8.31E-05 |
| chromosome, centromeric region | CC | 1.66E-06 | 2.08E-02 |
| ammonium transmembrane transporter | MF | 2.38E-06 | 9.71E-05 |
| system development | BP | 2.53E-06 | 2.75E-03 |
| regulation of inflammatory response | BP | 3.16E-06 | 2.86E-03 |
| regulation of interleukin-6 | BP | 3.97E-06 | 3.07E-03 |
| anatomical structure development | BP | 5.68E-06 | 3.85E-03 |
| RNA polymerase II core promoter | MF | 1.17E-05 | 1.47E-04 |
| macrophage differentiation | BP | 1.28E-05 | 7.72E-03 |
| antioxidant activity | MF | 1.30E-05 | 2.67E-04 |
| multicellular organismal development | BP | 1.46E-05 | 7.94E-03 |

**Table S3 Significantly enriched KEGG pathway (Top20)**

| **KEGG pathway** | **Count** | **P Value** | **FDR** |
| --- | --- | --- | --- |
| TGF-beta signaling pathway | 12 | 8.47E-04 | 2.44E-03 |
| cAMP signaling pathway | 19 | 9.39E-04 | 3.02E-03 |
| Ras signaling pathway | 11 | 1.01E-03 | 3.31E-03 |
| p53 signaling pathway | 16 | 1.02E-03 | 3.46E-03 |
| NF-κB signaling pathway | 10 | 1.46E-03 | 3.46E-03 |
| Apoptosis | 10 | 2.18E-03 | 4.13E-03 |
| Cytokine-cytokine receptor interaction | 17 | 2.68E-03 | 4.13E-03 |
| Apoptosis - multiple species | 15 | 2.74E-03 | 4.35E-03 |
| Melanogenesis | 16 | 3.08E-03 | 5.89E-03 |
| MAPK signaling pathway | 19 | 3.44E-03 | 6.71E-03 |
| JAK-STAT signaling pathway | 20 | 3.59E-03 | 8.95E-03 |
| cGMP-PKG signaling pathway | 9 | 4.67E-03 | 8.98E-03 |
| PI3K-Akt signaling pathway | 9 | 4.67E-03 | 9.08E-03 |
| Human T-cell leukemia virus 1 infection | 16 | 5.73E-03 | 9.08E-03 |
| mTOR signaling pathway | 13 | 7.08E-03 | 9.90E-03 |
| T cell receptor signaling pathway | 19 | 7.70E-03 | 1.01E-02 |
| ECM-receptor interaction | 9 | 9.97E-03 | 1.22E-02 |
| C-type lectin receptor signaling pathway | 15 | 1.02E-02 | 1.24E-02 |
| Melanoma | 13 | 1.39E-02 | 1.27E-02 |
| cell cycly | 8 | 1.58E-02 | 1.77E-02 |

**Table S4 Differential expression genes of M1 type macrophages between the CX3CL1 group and the control group (upregulated/downregulated Top10 gene)**

| **Gene ID** | **Gene Symbol** | **log_2_FC** | **p value** | **Type** |
| --- | --- | --- | --- | --- |
| 11074 | TRIM31 | 4.60 | 3.05E-12 | Up |
| 645754 | LGALS9DP | 4.32 | 3.40E-02 | Up |
| 60437 | CDH26 | 3.65 | 7.12E-05 | Up |
| 5166 | PDK4 | 3.54 | 4.34E-02 | Up |
| 8577 | TMEFF1 | 3.48 | 3.07E-02 | Up |
| 57412 | AS3MT | 3.42 | 1.80E-02 | Up |
| 2863 | GPR39 | 3.34 | 7.32E-04 | Up |
| 4647 | MYO7A | 3.31 | 4.14E-04 | Up |
| 316 | AOX1 | 3.25 | 1.58E-03 | Up |
| 6361 | CCL17 | 3.12 | 1.30E-04 | Up |
| 96626 | LIMS3 | -7.83 | 1.59E-07 | Down |
| 100288695 | LIMS4 | -6.85 | 4.31E-05 | Down |
| 440804 | RIMBP3B | -4.22 | 3.48E-02 | Down |
| 109245082 | PRAL | -3.86 | 1.05E-03 | Down |
| 100132815 | IPO5P1 | -3.60 | 1.39E-06 | Down |
| 641364 | SLC7A11-AS1 | -3.60 | 1.28E-05 | Down |
| 9052 | GPRC5A | -3.39 | 1.21E-04 | Down |
| 100874212 | MYCBP2-AS1 | -3.34 | 5.43E-04 | Down |
| 79094 | CHAC1 | -3.24 | 2.99E-02 | Down |
| 2911 | GRM1 | -3.17 | 1.60E-04 | Down |

**Table S5 Differential expression genes between CX3CL1 group and control group osteoclast (up/down regulation of Top10 gene)**

| **Gene ID** | **Gene Symbol** | **log_2_FC** | **p value** | **Type** |
| --- | --- | --- | --- | --- |
| 3507 | IGHM | 4.22 | 9.96E-10 | Up |
| 60437 | CDH26 | 4.18 | 1.14E-09 | Up |
| 51237 | MZB1 | 4.03 | 1.38E-09 | Up |
| 973 | CD79A | 3.88 | 1.53E-09 | Up |
| 83416 | FCRL5 | 3.85 | 2.02E-09 | Up |
| 5166 | PDK4 | 3.76 | 2.36E-09 | Up |
| 51303 | FKBP11 | 3.70 | 2.45E-09 | Up |
| 7852 | CXCR4 | 3.58 | 3.08E-09 | Up |
| 3492 | IGH | 3.47 | 3.71E-09 | Up |
| 6361 | CCL17 | 3.45 | 4.07E-09 | Up |
| 50486 | G0S2 | -2.46 | 9.21E-08 | Down |
| 283130 | SLC25A45 | -2.35 | 4.48E-07 | Down |
| 387357 | THEMIS | -2.18 | 1.17E-06 | Down |
| 23305 | ACSL6 | -2.10 | 1.19E-06 | Down |
| 96626 | LIMS3 | -2.08 | 1.73E-06 | Down |
| 100288695 | LIMS4 | -2.04 | 1.82E-06 | Down |
| 256380 | SCML4 | -1.95 | 3.07E-06 | Down |
| 4804 | NGFR | -1.94 | 3.36E-06 | Down |
| 3932 | LCK | -1.94 | 3.50E-06 | Down |
| 653188 | GUSBP3 | -1.94 | 3.52E-06 | Down |

**Table S6 Intersection differentially expressed genes differentially expressed genes (upregulated/downregulated Top10 gene)**

| Gene ID | Gene Symbol | log_2_FC | p value | Type |
| --- | --- | --- | --- | --- |
| 3514 | IGKC | 4.95 | 4.43E-07 | Up |
| 286530 | P2RY8 | 4.32 | 4.40E-07 | Up |
| 9333 | TGM5 | 4.06 | 4.39E-07 | Up |
| 83888 | FGFBP2 | 4.05 | 4.35E-07 | Up |
| 3501 | IGHG2 | 4.02 | 4.34E-07 | Up |
| 301 | ANXA1 | 3.95 | 4.33E-07 | Up |
| 6910 | TBX5 | 3.88 | 4.31E-07 | Up |
| 8302 | KLRC4 | 3.81 | 4.3E-07 | Up |
| 3221 | HOXC4 | 3.76 | 4.24E-07 | Up |
| 11082 | ESM1 | 3.70 | 4.24E-07 | Up |
| 6457 | SH3GL3 | -5.83 | 4.07E-09 | Down |
| 55503 | TRPV6 | -5.23 | 4.03E-09 | Down |
| 3881 | KRT31 | -4.98 | 3.99E-09 | Down |
| 85004 | RERG | -4.90 | 3.91E-09 | Down |
| 56302 | TRPV5 | -4.87 | 3.88E-09 | Down |
| 29895 | MYLPF | -4.79 | 3.84E-09 | Down |
| 3798 | KIF5A | -4.69 | 3.82E-09 | Down |
| 56924 | PAK6 | -4.69 | 3.82E-09 | Down |
| 255275 | MYADML2 | -4.68 | 3.73E-09 | Down |

**Table S7 Significantly enriched GO pathway (Top30)**

| **GO Term** | **GO Category** | **p value** | **FDR** |
| --- | --- | --- | --- |
| cell adhesion | BP | 5.68E-19 | 3.19E-14 |
| biological adhesion | BP | 6.60E-19 | 3.67E-10 |
| atrial cardiac muscle cell action potential | BP | 2.75E-15 | 4.09E-10 |
| atrial cardiac muscle cell to AV node cell signaling | BP | 7.04E-15 | 8.68E-10 |
| regulation of atrial cardiac muscle cell membrane | BP | 8.64E-15 | 2.53E-09 |
| atrial cardiac muscle cell membrane repolarization | BP | 8.64E-15 | 2.69E-09 |
| atrial cardiac muscle cell to AV node cell | BP | 1.06E-13 | 2.77E-09 |
| immune system process | BP | 2.26E-13 | 5.05E-09 |
| chemokine-mediated signaling pathway | BP | 5.28E-13 | 5.69E-09 |
| cell periphery | CC | 9.48E-13 | 3.22E-06 |
| plasma membrane | CC | 9.87E-13 | 1.56E-05 |
| response to chemokine | BP | 3.26E-12 | 5.89E-09 |
| cellular response to chemokine | BP | 4.77E-12 | 7.75E-09 |
| integral component of plasma membrane | CC | 6.98E-12 | 1.58E-05 |
| cell-cell signaling involved in cardiac conduction | BP | 9.97E-12 | 2.76E-08 |
| intrinsic component of plasma membrane | CC | 1.30E-11 | 2.50E-04 |
| membrane repolarization during action potential | BP | 3.22E-11 | 4.08E-08 |
| membrane repolarization during cardiac muscle cell | BP | 7.01E-11 | 4.88E-08 |
| cardiac muscle cell membrane repolarization | BP | 7.37E-11 | 5.16E-08 |
| cellular response to cytokine stimulus | BP | 2.37E-10 | 6.82E-08 |
| regulation of multicellular organismal process | BP | 2.62E-10 | 7.68E-08 |
| regulation of cardiac muscle cell membrane | BP | 4.33E-10 | 1.04E-07 |
| external encapsulating structure organization | BP | 7.95E-10 | 1.61E-07 |
| negative regulation of mast cell activation | BP | 8.04E-10 | 2.17E-07 |
| extracellular matrix | CC | 1.96E-09 | 4.52E-04 |
| external encapsulating structure | CC | 2.86E-09 | 7.61E-04 |
| basal part of cell | CC | 6.54E-09 | 8.24E-04 |
| integrin binding | MF | 3.25E-08 | 4.99E-06 |
| growth factor binding | MF | 4.62E-08 | 1.17E-05 |
| neurotrophin binding | MF | 8.95E-08 | 1.42E-05 |

**Table S8 Significantly enriched KEGG pathway (Top20)**

| **KEGG pathway** | **Count** | **p value** | **FDR** |
| --- | --- | --- | --- |
| NF-κB signaling pathway | 14 | 3.71E-09 | 2.30E-07 |
| Cytokine-cytokine receptor interaction | 12 | 1.18E-08 | 4.33E-06 |
| PI3K-Akt signaling pathway | 13 | 3.64E-07 | 6.78E-05 |
| Wnt signaling pathway | 11 | 3.89E-07 | 1.97E-04 |
| Hematopoietic cell lineage | 10 | 3.58E-06 | 5.54E-04 |
| Axon guidance | 9 | 3.93E-06 | 1.62E-03 |
| Small cell lung cancer | 7 | 1.46E-05 | 1.99E-03 |
| Cholinergic synapse | 9 | 1.88E-05 | 3.35E-03 |
| Chemokine signaling pathway | 8 | 3.01E-05 | 5.35E-03 |
| Jak-STAT signaling pathway | 9 | 3.12E-05 | 5.61E-03 |
| Leukocyte transendothelial migration | 11 | 3.52E-05 | 1.01E-02 |
| Estrogen signaling pathway | 10 | 4.21E-05 | 1.04E-02 |
| ECM-receptor interaction | 8 | 6.24E-05 | 1.11E-02 |
| Bladder cancer | 12 | 6.55E-05 | 1.14E-02 |
| Tryptophan metabolism | 11 | 1.03E-04 | 1.24E-02 |
| Glycosaminoglycan biosynthesis - heparan | 6 | 1.48E-04 | 1.25E-02 |
| Fluid shear stress and atherosclerosis | 9 | 2.74E-04 | 2.54E-02 |
| Protein digestion and absorption | 5 | 3.48E-04 | 3.04E-02 |
| mTOR signaling pathway | 10 | 6.91E-04 | 4.13E-02 |
| Hippo signaling pathway | 9 | 7.24E-04 | 4.81E-02 |

**Table S9 Changes in General Conditions of Mice (n=10)**

| Group | Food intake | Water intake | Mentality | Hair Gloss |
| --- | --- | --- | --- | --- |
| Control |  |  |  |  |
| Model | **‑ ‑ ‑** | + + + | **‑ ‑ ‑** | **‑ ‑ ‑** |

Note: "+" indicates increase, excitement, and good; "-" indicates a decrease, decline, or deterioration.
